# PEDIA: prioritization of exome data by image analysis

**DOI:** 10.1038/s41436-019-0566-2

**Published:** 2019-06-05

**Authors:** Tzung-Chien Hsieh, Martin A. Mensah, Jean T. Pantel, Dione Aguilar, Omri Bar, Allan Bayat, Luis Becerra-Solano, Heidi B. Bentzen, Saskia Biskup, Oleg Borisov, Oivind Braaten, Claudia Ciaccio, Marie Coutelier, Kirsten Cremer, Magdalena Danyel, Svenja Daschkey, Hilda David Eden, Koenraad Devriendt, Sandra Wilson, Sofia Douzgou, Dejan Đukić, Nadja Ehmke, Christine Fauth, Björn Fischer-Zirnsak, Nicole Fleischer, Heinz Gabriel, Luitgard Graul-Neumann, Karen W. Gripp, Yaron Gurovich, Asya Gusina, Nechama Haddad, Nurulhuda Hajjir, Yair Hanani, Jakob Hertzberg, Konstanze Hoertnagel, Janelle Howell, Ivan Ivanovski, Angela Kaindl, Tom Kamphans, Susanne Kamphausen, Catherine Karimov, Hadil Kathom, Anna Keryan, Alexej Knaus, Sebastian Köhler, Uwe Kornak, Alexander Lavrov, Maximilian Leitheiser, Gholson J. Lyon, Elisabeth Mangold, Purificación Marín Reina, Antonio Martinez Carrascal, Diana Mitter, Laura Morlan Herrador, Guy Nadav, Markus Nöthen, Alfredo Orrico, Claus-Eric Ott, Kristen Park, Borut Peterlin, Laura Pölsler, Annick Raas-Rothschild, Linda Randolph, Nicole Revencu, Christina Ringmann Fagerberg, Peter Nick Robinson, Stanislav Rosnev, Sabine Rudnik, Gorazd Rudolf, Ulrich Schatz, Anna Schossig, Max Schubach, Or Shanoon, Eamonn Sheridan, Pola Smirin-Yosef, Malte Spielmann, Eun-Kyung Suk, Yves Sznajer, Christian T. Thiel, Gundula Thiel, Alain Verloes, Irena Vrecar, Dagmar Wahl, Ingrid Weber, Korina Winter, Marzena Wiśniewska, Bernd Wollnik, Ming W. Yeung, Max Zhao, Na Zhu, Johannes Zschocke, Stefan Mundlos, Denise Horn, Peter M. Krawitz

**Affiliations:** 1 Institute of Genomic Statistics and Bioinformatics, University of Bonn, Bonn, Germany; 2 Charité – Universitätsmedizin Berlin, corporate member of Freie Universität Berlin, Humboldt-Universität zu Berlin, and Berlin Institute of Health, Institute of Medical Genetics and Human Genetics, Berlin, Germany; 3 Berlin Institute of Health (BIH), Berlin, Germany; 4 Centro de Cáncer de Mama, Tecnológico de Monterrey, Monterrey, Mexico; 5 FDNA Inc., Boston, MA USA; 6 Rigshospitalet, Department of Neurology, Copenhagen, Denmark; 7 Unidad de Investigación Médica en Medicina Reproductiva, Mexico City, Mexico; 8 Centre for Medical Ethics, Faculty of Medicine and the Norwegian Research Center for Computers and Law, Faculty of Law, University of Oslo, Oslo, Norway; 9 CeGaT GmbH, Tübingen, Germany; 10 Faculty of Medicine, Department of Medical Genetics, University of Oslo, Blindern, Oslo, Norway; 11 Developmental Neurology Unit, Fondazione IRCCS Istituto Neurologico Carlo Besta, Milan, Italy; 12 Department of Human Genetics, University Hospital of Bonn, Bonn, Germany; 13 Medical Faculty, Heinrich Heine University Düsseldorf, Düsseldorf, Germany; 14 Department of Human Genetics, KU Leuven, Leuven, Belgium; 15 Department of Human Genetics, University of Hamburg, Hamburg, Germany; 16 Manchester Centre for Genomic Medicine, St Mary’s Hospital, Central Manchester University Hospitals NHS Foundation Trust Manchester Academic Health Sciences Centre, Manchester, United Kingdom; 17 School of Biological Sciences, Division of Evolution and Genomic Sciences, University of Manchester, Manchester, United Kingdom; 18 Division of Human Genetics, Medical University of Innsbruck, Innsbruck, Austria; 19 Center for Genomics and Transcriptomics, Eberhard Karls University of Tübingen, Tübingen, Germany; 20 A. I. duPont Hospital for Children, Wilmington, DE USA; 21 National Research and Applied Medicine Centre ‘Mother and Child’’, Minsk, Belarus; 22 Lineagen, Salt Lake City, Utah USA; 23 Clinical Genetics Unit, AUSL-IRCCS Reggio Emilia, Reggio Emilia, Italy; 24 Center for Chronically Sick Children (Sozialpädiatrisches Zentrum, SPZ), Charité - Universitätsmedizin Berlin, Berlin, Germany; 25 GeneTalk, Bonn, Germany; 26 University Hospital Magdeburg, Magdeburg, Germany; 27 Children’s Hospital of Los Angeles, Los Angeles, CA USA; 28 Department of Pediatrics, Medical University of Sofia, Sofia, Bulgaria; 29 Charité – Universitätsmedizin Berlin, corporate member of Freie Universität Berlin, Humboldt-Universität zu Berlin, and Berlin Institute of Health, NeuroCure Clinical Research Center, Berlin, Germany; 30 Research Institute of Medical Genetics of Russian Academy of Medical Sciences, Moscow, Russian Federation; 31 Stanley Institute for Cognitive Genomics, Cold Spring Harbor Laboratory, Woodbury, New York USA; 32 Institute of Human Genetics, University of Bonn, Bonn, Germany; 33 Hospital General Universitario De Valencia, Valencia, Spain; 34 Hospital General De Requena, Servicio Pediatría, Spain; 35 University Hospital Leipzig, Leipzig, Germany; 36 Hospital Universitario Miguel Servet, Zaragoza, Spain; 37 Azienda Ospedaliera Universitaria Senese, Siena, Italy; 38 Department of Pediatrics and Neurology, University of Colorado School of Medicine, Colorado Aurora, USA; 39 Clinical Institute of Medical Genetics, University Medical Centre Ljubljana, Ljubljana, Slovenia; 40 The Danek Gertner Institute of Human Genetics, Sheba Medical Center, Tel-Hashomer, Israel; 41 Center for Human Genetics, University Hospital, Université Catholique de Louvain, Brussels, Belgium; 42 Odense University Hospital, Odense, Denmark; 43 The Jackson Laboratory for Genomic Medicine, Farmington, CT USA; 44 School of Medicine, University of Leeds, Leeds, United Kingdom; 45 Genomic Bioinformatics Laboratory, Department of Molecular Biology, Ariel University, Ariel, Israel; 46 Center for Prenatal Diagnosis and Human Genetics, Berlin, Germany; 47 Cliniques universitaires Saint Luc UCL, Bruxelles, Belgium; 48 Institute of Human Genetics, Friedrich-Alexander-Universität Erlangen-Nürnberg FAU, Erlangen, Erlangen, Germany; 49 Hopital Robert Debré, Paris, France; 50 Center for Human Genetics and Laboratory Diagnostics Dr. Klein, Dr. Rost and Colleagues, Martinsried, Germany; 51 Poznañ University of Medical Sciences, Poznañ, Poland; 52 University Medical Center Göttingen, Göttingen, Germany

**Keywords:** deep learning, computer vision, dysmorphology, variant prioritization, exome diagnostics

## Abstract

**Purpose:**

Phenotype information is crucial for the interpretation of genomic variants. So far it has only been accessible for bioinformatics workflows after encoding into clinical terms by expert dysmorphologists.

**Methods:**

Here, we introduce an approach driven by artificial intelligence that uses portrait photographs for the interpretation of clinical exome data. We measured the value added by computer-assisted image analysis to the diagnostic yield on a cohort consisting of 679 individuals with 105 different monogenic disorders. For each case in the cohort we compiled frontal photos, clinical features, and the disease-causing variants, and simulated multiple exomes of different ethnic backgrounds.

**Results:**

The additional use of similarity scores from computer-assisted analysis of frontal photos improved the top 1 accuracy rate by more than 20–89% and the top 10 accuracy rate by more than 5–99% for the disease-causing gene.

**Conclusion:**

Image analysis by deep-learning algorithms can be used to quantify the phenotypic similarity (PP4 criterion of the American College of Medical Genetics and Genomics guidelines) and to advance the performance of bioinformatics pipelines for exome analysis.

## INTRODUCTION

Worldwide, more than half a million children born per year have a rare genetic disorder that is suitable for diagnostic evaluation by exome sequencing. This test’s unprecedented diagnostic yield is contrasted by the time requirement for variant interpretation. Making phenotypic information—the observable, clinical presentation—computer-readable is key to solving this problem and important for providing clinicians with a much-needed tool for diagnosing genetic syndromes.^1^

To date, the most advanced exome prioritization algorithms combine deleteriousness scores for variants with semantic similarity searches of the clinical description of a patient.^2^ The Human Phenotype Ontology (HPO) has become the *lingua franca* for this purpose.^3^ However, a facial gestalt for which no term exists and that is simply described as "characteristic" for a certain disease is not suitable for these computational approaches.

Beyond language, capturing indicative patterns through deep-learning approaches has recently gained attention in assessing facial dysmorphism.^4,5^ Artificial neural networks measure the similarities of patient photos to hundreds of disease entities. We hypothesized that results of this next-generation phenotyping tool could be used similarly to deleteriousness scores on the molecular level. This would enable us to transition from the dichotomous PP4 criterion “matching phenotype” in the American College of Medical Genetics and Genomics (ACMG) guidelines for variant interpretation to a quantifiable one.^6,7^

We therefore developed an approach to interpret sequence variants integrating results from the next-generation phenotyping tool DeepGestalt. By this means the clinical presentation of an individual is not only assessed by a human expert clinician, but also by using an artificial intelligence approach on the basis of frontal photographs. In short, we call this approach prioritization of exome data by image analysis (PEDIA).

## MATERIALS AND METHODS

We compiled a cohort comprising 679 individuals with frontal facial photographs and clinical features documented in HPO terminology.^3^ The diagnoses of all individuals have previously been confirmed molecularly and are suitable for analysis by exome sequencing. In total, the cohort covers 105 different monogenic syndromes linked to 181 different genes. Of the individuals in this cohort, 446 were published and 233 have not been previously reported (see PMID column in Supplementary Table 1).

The study was approved by the ethics committees of the Charité–Universitätsmedizin Berlin and of the University Hospital Bonn. Written informed consent was given by the patients or their guardians, including permission to publish photographs. Easy to understand, transparent information with both text and illustrations about the pattern recognition in our algorithm that processes personal data in the form of 2D portrait photographs can be found at https://www.pedia-study.org/documents. Through technical and organizational measures (privacy by design), we process the photos and the data obtained from them in the least identifiable manner necessary for achieving the purpose. This respects the data minimization principle of data being adequate, relevant, and limited.

In addition to the PEDIA data set, we analyzed a subset of the DeepGestalt study. By removing disorders that are confirmed by tests other than exome sequencing, such as Down syndrome (Supplementary Table 2), we ended up with 260 of 329 cases from the DeepGestalt set.^5^

The facial images were analyzed with DeepGestalt, a deep convolutional neural network trained on more than 17,000 patient images.^5^ The results of this analysis are gestalt scores that quantify the similarity to 216 different rare phenotypes per individual. These vectors can also be used to identify duplicates in the DeepGestalt training set and test set without the need to access the original photos. To avoid overfitting, we excluded all cases of the PEDIA cohort from a DeepGestalt model that we used for benchmarking. It is noteworthy that the version of DeepGestalt available at Face2Gene will not yield the same results when photos of the PEDIA cohort are reanalyzed because it is built as a framework that aims to learn from every solved case.

**Fig. 1:**
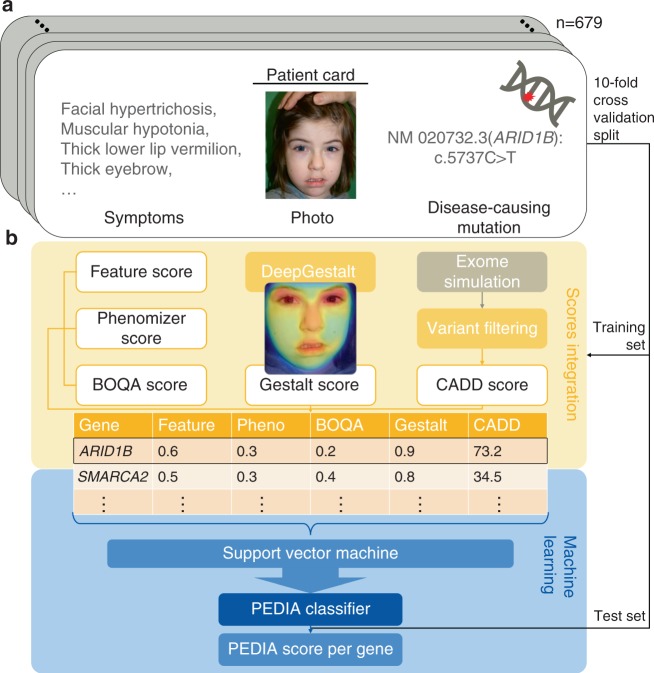
**Prioritization of exome data by image analysis (PEDIA): cohort and classification approach.** (**a**) Clinical features, facial photograph, and pathogenic variant of one individual of the PEDIA cohort. In total the cohort consists of 679 cases with monogenic disorders that are suitable for a diagnostic workup by exome sequencing. (**b**) Clinical features, images, and exome variants were evaluated separately and integrated to a single score by a machine learning approach. The disease-causing gene is shown at the top of the list.

In addition to the image analysis, we performed semantic similarity searches with the annotated HPO terms by three different tools: Feature Match (FDNA), Phenomizer, and Bayesian Ontology Querying for Accurate Comparisons (BOQA).^8,9^ HPO terms for all published cases as well as the clinical notes in the electronic health records were independently extracted by two data curators. All terms that did not occur in both lists were revisited by a third curator (see Fig. 1a and Supplementary Table 1). The similarity scores from image analysis as well as semantic similarity searches were mapped to genes by mim2gene and morbidmap from OMIM.^10^ If there were several syndromes linked to a gene, the highest gestalt and feature scores were selected for this gene.

Exome sequencing data was not available for the vast majority of cases. Therefore, we spiked in the disease-causing variant of each case into randomly selected exomes of healthy individuals of different ethnicities from the 1000 Genomes Project.^11^ All sequence variants were then filtered as described by Wright et al. and scored for deleteriousness with CADD.^12,13^ Per gene, the variant with the highest CADD score was used, regardless of the genotype. This heuristic was chosen to maximize the sensitivity also for compound heterozygous cases where the second hit in a recessive disease gene achieves only a relatively low CADD score.

For each case this procedure resulted in a table with rows for genes and the five different scores in the columns (Fig. 1b). All five scores per line as well as the Boolean label disease gene “true” or “false” (i.e., the vector) were used to train a classifier that yields a single value per gene, the PEDIA score, that can be used for prioritization (Fig. 1b). A detailed description of preprocessing and filtering, as well as all the annotated data, can be found in our code repository.

We used a support vector machine (SVM) to prioritize the genes based on the five scores for each case. To benchmark our approach, we performed tenfold cross-validation. First, we split the PEDIA cohort into ten groups, ensuring that a certain disease gene was included only in one of ten groups. By this means, we avoided overfitting, in case the same disease-causing variant occurred in two different individuals (Supplementary Fig. 1). We used a linear kernel on the five scores to train the SVM and selected the hyperparameter C in the range from 2^−6^ to 2^12^ by performing internal fivefold cross-validation on the training set. The C with the highest top 1 accuracy was selected for training a linear SVM. We further benchmarked the performance of each case in the test set with this model. The distance of each gene to the hyperplane—defined as the PEDIA score—was used to rank the genes for the case. If the disease-causing gene was at the first position, we called it a top 1 match, or if it was among the first ten genes, we considered it a top 10 match.

For the 260 cases from the DeepGestalt publication test set, where exome diagnostics would be applicable, we randomly selected cases from the PEDIA cohort with the same diagnosis and added the CADD and the feature scores per case (see column C in Supplemental Table 1). The cases in the PEDIA cohort with the same pathogenic variant as already assigned to the DeepGestalt test set were removed from the training set. Then we trained the classifier on the PEDIA cohort and tested it on the DeepGestalt publication test set. The experiment was repeated ten times with random selection. By this means we studied how the publicly available portraits of the DeepGestalt test set would improve the performance when used in exome analysis with the PEDIA approach. However, it has to be emphasized that both approaches solve different multiclass classification problems (MCPs), the first tool operating on phenotypes and the second on genes. The difficulty of the task is not only characterized by the number of classes and the distinguishability of the different entities but also by the information available for the classification. For both MCPs the maximum number of classes can be estimated from OMIM by querying with the HPO term “abnormal facial shape”, yielding around 700 disorders and genes with disease-causing variants. As there is additional and nonredundant information available from the molecular level for PEDIA, it achieves better top 1 and top 10 accuracies.

## Code availability

All training data as well as the classifier are available at https://github.com/PEDIA-Charite/PEDIA-workflow. The trained PEDIA model is provided as a service that is ready to use at https://pedia-study.org.

## RESULTS

The performance of a prioritization tool can be assessed by the proportion of cases for which the correct diagnosis or disease gene is placed at the first position or among the first ten suggestions (top 1 and top 10 accuracy). The composition of the test set has an influence on the accuracy because some disease phenotypes are easier to recognize, and some gene variants are more readily identified as deleterious. The setup of the PEDIA cohort, which is comprehensively documented in the Supplementary Appendix, therefore aims at emulating the whole spectrum of cases that could be analyzed with DeepGestalt and diagnosed by exome sequencing.

When only CADD scores are used for variant ranking, the disease-causing gene is in the top 10 in less than 45% of all tested cases. The top 10 accuracy increases up to 63–94%, when different semantic similarity scores based on HPO feature annotations are included (Supplementary Table 3).

**Fig. 2:**
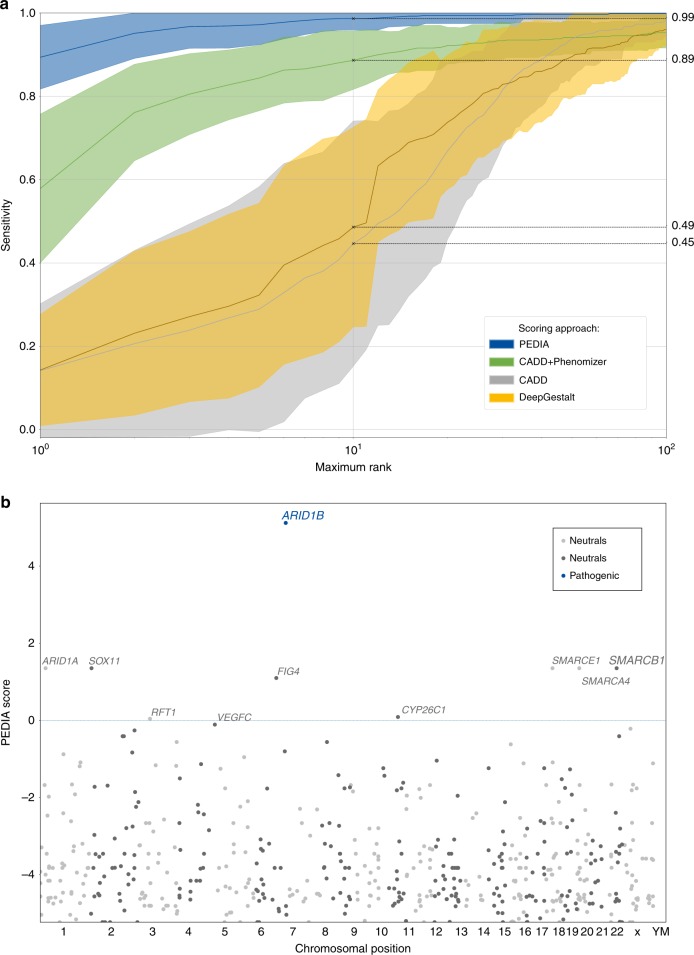
**Performance readout and visualization of test results for a representative prioritization of exome data by image analysis (PEDIA) case**. (**a**) For each case the exome variants are ordered according to four different scoring approaches, solely by a molecular deleteriousness score (CADD), by a score from image analysis (DeepGestalt), by a combination of a molecular deleteriousness score and a clinical feature–based semantic similarity score (CADD+Phenomizer), or the PEDIA score that includes all three levels of evidence. The sensitivity of the prioritization approach depends on the number of genes that are considered in an ordered list. The top 1 and top 10 accuracy rates correspond to the intersection of the curves at maximum rank 1 and 10. Note that for benchmarking DeepGestalt on the gene level, syndrome similarity scores first have to be mapped to the gene level, resulting in a lower performance compared with the readout on a phenotype level, due to heterogeneity. The area under the curve is largest for PEDIA scoring. (**b**) The disease-causing gene of the case depicted in Fig. 1 achieves the highest PEDIA score and molecularly confirms the diagnosis of Coffin–Siris syndrome. Other genes associated with similar phenotypes, such as Nicolaides–Baraitser syndrome, also achieved high scores for gestalt but not for variant deleteriousness.

The additional information from frontal photos of cases pushes the correct disease gene to the top 10 in 99% of all PEDIA cases (Fig. 2a). Particularly striking is the performance gain for the top 1 accuracy rate from 36–74% without DeepGestalt scores to 86–89% including the scores from image analysis (Supplementary Table 3).

The distribution of the PEDIA scores does not differ using exomes with different ethnic backgrounds (Supplementary Fig. 2).

Although the top 10 accuracies of DeepGestalt scoring on the phenotype level and PEDIA scoring on the gene level cannot be compared directly, both approaches operate on a similar number of classes (Fig. 2). Adding suitable molecular information to 260 cases from the DeepGestalt publication test set confirms our results in the PEDIA cohort by achieving a top 10 accuracy rate of 99% (Supplementary Table 2).

The value of a frontal photograph is demonstrated by a case with Coffin–Siris syndrome (shown in Fig. 1): the characteristic facial features are relatively mild, so the correct diagnosis is only listed as the third suggestion by DeepGestalt. Among all the variants encountered in the exome, the disease-causing gene *ARID1B* would only achieve rank 27, if scored by the molecular information alone. However, combined with the phenotypic information, the PEDIA approach lists this gene as the first candidate (Fig. 2b).

Although the diagnosis of the illustrated case could be molecularly confirmed by a directed single-gene test in other instances where the facial gestalt is more indicative, syndromic disorders often puzzle clinicians due to their high phenotypic variability. In the Deciphering Developmental Disorders (DDD) project many syndromes were diagnosed only after exome sequencing.^14^ Still, the top 10 accuracy rate of 49% that DeepGestalt can achieve for phenotypes linked to genes is impressive (Fig. 2a). The contribution from the different sources of evidence to the PEDIA score is also reflected by the relative weight of the deleteriousness of the pathogenic variant (0.44), all feature-based scores combined (0.25), and the results from image analysis by DeepGestalt (0.31) that can be derived from a linear SVM model. The information contained in a frontal photograph of a patient therefore goes beyond what clinical terms can capture. The top 1 and top 10 accuracies are reported for all combinations of scores in the Supplementary Table 3.

## DISCUSSION

The guidelines for variant classification in the laboratory follow a qualitative heuristic that combines distinct types of evidence (functional, population, phenotype, etc.). Interestingly, it is also compatible with Bayesian statistics^7^ and the advantage of such a framework is that continuous evidence types can be integrated into the classification system. While in silico predictions about a variant’s pathogenicity have a relatively long history in bioinformatics and machine learning, the quantification of phenotypic raw data such as facial images with artificial intelligence systems has just begun: the PEDIA approach uses scores from DeepGestalt for gene prioritization in combination with quantitative scores from the molecular level in Mendelian disorders identifiable by exome sequencing.

Interestingly, the ethnicity, which affects the number of variant calls or the deleterious variant load, had minor influence on the performance of PEDIA. Although the total number of variants detected by reference-guided sequencing in individuals of African descent is considerably higher than in individuals of European or Asian descent, the distribution of the CADD scores for rare variants is comparable (Supplementary Figs. 3, 4). That means the rank that a gene achieves due to the molecular score and the corresponding scores from the phenotypic information is hardly affected by the background population (Supplementary Fig. 2).

With regard to the routine use in the laboratory we have learned three important lessons from specific subgroups or cases achieving lower PEDIA ranks:

1. Although DeepGestalt, the convolutional neural network used for image analysis, has been pretrained on real-world uncontrolled 2D images, patient photographs that were not frontal, of low resolution, had poor lightening and contrast, or contained artifacts such as glasses, yielded lower gestalt scores for the searched disorder. In one use case envisioned for PEDIA, the human expert in the lab will only receive the similarity scores from DeepGestalt, but not the original photograph. In this setting it is not clear whether low scores originate from a low-quality photograph or whether there is little dysmorphic signal indicative of a syndromic disorder. This potential problem could be addressed by providing gestalt scores from additional photographs.

2. Particularly rare diseases or recently described disorders, for which the classifier’s representation is based on a smaller training set, show a lower performance, even if experienced dysmorphologists would consider them highly distinguishable. In a recent publication by Duddin-Byth et al. the machine learning approach showed the lowest accuracy for the disorder with the smallest number of training cases; however, so did humans.^15^

3. Disease-causing variants in genes that interact in a molecular pathway often result in highly similar phenotypes that are organized as series in OMIM and modeled as a single entity by DeepGestalt. Often there are subtle gene-specific differences in the gestalt and modeling the entire phenotypic series by a single class is not the theoretical optimum achievable with more cases.^16,17^ This will especially diminish the performance of genes less frequently mutated in a molecular pathway. This is exemplified in the PEDIA cohort by Hyperphosphatasia with Mental Retardation Syndrome (HPMRS), where the least frequently mutated gene, *PGAP2*, shows the lowest performance. Likewise, this applies to microdeletion syndromes that can also be caused by pathogenic variants in single genes, such as Smith–Magenis syndrome, or an atypical clinical presentation with Kabuki syndrome (see e.g., case IDs 246245 and 204233 in Supplementary Table 1).^18^

It is noteworthy that these shortcomings are mainly due to the limited training data for these particular genes and that they will most likely be overcome by more molecularly confirmed cases. DeepGestalt and PEDIA are therefore built as frameworks that will be improved continuously with additional data. In general, the use of artificial intelligence in medical sciences raises new or exacerbates existing ethical and legal issues as repositories of combined genotype and phenotype data become crucial for the machine learning community.^19,20^ Sharing portrait photos of individuals with rare diseases can be accomplished within the scope of even the most elaborate data privacy laws, such as the European Union General Data Protection Regulation 2016/679 (GDPR). The GDPR not only ensures the protection of individuals, but also the free movement of personal data, inter alia, for scientific research purposes.^21^

The interpretation of genetic variants is greatly facilitated by sequencing additional family members. Analogously, we hypothesize that the signal-to-noise ratio of next-generation phenotyping technologies can further be improved by including unaffected siblings or parents in the analysis.

We include and strive to include a wide variety of ethnicities, but European backgrounds are currently best represented, leading to best performance for this population. As the data set expands further, the algorithm will improve for currently underrepresented ethnicities.

Assistance with diagnosis of rare genetic disorders is highly valuable to clinicians, and by extension to the patients themselves and their families. Especially in inconclusive cases with findings of unknown clinical significance, additional evidence from computer-assisted analysis of medical imaging data could be a decisive factor.^13^

In conclusion, the PEDIA study documents that exome variant interpretation benefits from computer-assisted image analysis of facial photographs. By including similarity scores from DeepGestalt, we improved the top 10 accuracy rate significantly compared with state-of-the-art algorithms. Artificial intelligence–driven pattern recognition of frontal facial patient photographs is therefore an example of next-generation phenotyping technology that has proven its clinical value for the interpretation of next-generation sequencing data.^22^

## Supplementary information

Supplementary_Material

SupplementaryTable 1
