# Supplementary material for: PEDIA: prioritization of exome data by image analysis
Source: Genet Med. 2019 Jun 5;21(12):2807–14. doi: 10.1038/s41436-019-0566-2 (PMC6892739; doi:10.1038/s41436-019-0566-2)
Supplement: Supplementary file 1 — Supplementary_Material [file 41436_2019_566_MOESM1_ESM.pdf]

Prioritization of Exome Data by Image Analysis Supplementary Material

Hsieh, Tzung-Chien *[1,2], Mensah, Martin-Atta *[2,3], Pantel, Jean Tori[2,3], Aguilar, Dione[4], Bar, Omri[5], Bayat, Allan[6], Becerra-Solano, Luis[7], Bentzen, Heidi Beate[8], Biskup, Saskia[9], Borisov, Oleg[1], Braaten, Oivind[10], Ciaccio, Claudia[11], Coutelier, Marie[2], Cremer, Kirsten[12], Danyel, Magdalena[2], Daschkey, Svenja[13], David Eden, Hilda[5], Devriendt, Koenraad[14], Dölken, Sandra[15], Douzgou, Sofia[16,17], Đukić, Dejan[1], Ehmke, Nadja[2], Fauth, Christine[18], Fischer-Zirnsak, Björn[2], Fleischer, Nicole[5], Gabriel, Heinz[19], Graul-Neumann, Luitgard[2], Gripp, Karen W.[20], Gurovich, Yaron[5], Gusina, Asya[21], Haddad, Nechama[2], Hajjir, Nurulhuda[2], Hanani, Yair[5], Hertzberg, Jakob[2], Hoertnagel, Konstanze[9], Howell, Janelle[22], Ivanovski, Ivan[23], Kaindl, Angela[24], Kamphans, Tom[25], Kamphausen, Susanne[26], Karimov, Catherine[27], Kathom, Hadil[28], Keryan, Anna[27], Knaus, Alexej[1], Köhler, Sebastian[29], Kornak, Uwe[2], Lavrov, Alexander[30], Leitheiser, Maximilian[2], Lyon, Gholson J.[31], Mangold, Elisabeth[32], MarÃŋn Reina, PurificaciÃşn[33], Martinez Carrascal, Antonio[34], Mitter, Diana[35], Morlan Herrador, Laura[36], Nadav, Guy[5], Nöthen, Markus[12], Orrico, Alfredo[37], Ott, Claus-Eric[2], Park, Kristen[38], Peterlin, Borut[39], Pölsler, Laura[18], Raas-Rothschild, Annick[40], Randolph, Linda[27], Revencu, Nicole[41], Ringmann Fagerberg, Christina[42], Robinson, Peter Nick[43], Rosnev, Stanislav[2], Rudnik, Sabine[18], Rudolf, Gorazd[39], Schatz, Ulrich[18], Schossig, Anna[18], Schubach, Max[3], Shanoon, Or[5], Sheridan, Eamonn[44], Smirin-Yosef, Pola[45], Spielmann, Malte[2], Suk, Eun-Kyung[46], Sznajer, Yves[47], Thiel, Christian Thomas[48], Thiel, Gundula[46], Verloes, Alain[49], Vrecar, Irena[39], Wahl, Dagmar[50], Weber, Ingrid[18], Winter, Korina[2], Wiśniewska, Marzena[51], Wollnik, Bernd[52], Yeung, Ming Wai[1], Zhao, Max[2], Zhu, Na[2], Zschocke, Johannes[18], Mundlos, Stefan[2], Horn, Denise[2], and Krawitz, Peter[1,+]

[1]Institute of Genomic Statistics and Bioinformatics, University of Bonn, Bonn, Germany
[2]Charité Universitätsmedizin Berlin, corporate member of Freie Universität Berlin, Humboldt-Universität zu Berlin, and Berlin Institute of Health, Institute of Medical Genetics and Human Genetics, Berlin, Germany
[3]Berlin Institute of Health (BIH), Anna-Louisa-Karsch 2, 10178 Berlin, Germany
[4]Monterrey Institute of Technology and Higher Education, Mexico
[5]FDNA Inc., Boston Massachusetts, United States
[6]Rigshospitalet, Department of Neurology, Copenhagen, Denmark
[7]Unidad de Investigación Médica en Medicina Reproductiva, Mexico
[8]Centre for Medical Ethics, Faculty of Medicine and the Norwegian Research Center for Computers and Law, Faculty of Law, University of Oslo, Oslo, Norway
[9]CeGaT GmbH, Tübingen, Germany
[10]Faculty of Medicine, Department of Medical Genetics, University of Oslo, Blindern, Oslo, Norway
[11]University of Milan, Milan, Italy
[12]Department of Human Genetics, University Hospital of Bonn, Bonn, Germany
[13]Heinrich Heine University Düsseldorf, Düsseldorf, Germany
[14]Catholic University Leuven, Leuven, Belgium
[15]University of Hamburg, Hamburg, Germany
[16]Manchester Centre for Genomic Medicine, St Mary's Hospital, Central Manchester University Hospitals NHS Foundation Trust Manchester Academic Health Sciences Centre, Manchester, United Kingdom
[17]School of Biological Sciences, Division of Evolution and Genomic Sciences, University of Manchester, Manchester, United Kingdom
[18]Division of Human Genetics, Medical University of Innsbruck, Innsbruck, Austria
[19]University of Tübingen, Tübingen, Germany
[20]A. I. duPont Hospital for Children, Wilmington, United States
[21]National Research and Applied Medicine Centre "Mother and Child", Belarus
[22]Lineagen, United States

[23]Santa Maria Nuova Hospital, Italy
[24]Center for Chronically Sick Children (Sozialpädiatrisches Zentrum, SPZ), Charité - Universitätsmedizin Berlin, Berlin, Germany
[25]GeneTalk, Bonn, Germany
[26]University Hospital Magdeburg, Magdeburg, Germany
[27]Children's Hospital of Los Angeles, Los Angeles, United States
[28]Medical University of Sofia, Sofia, Bulgaria
[29]Charité âĂŞ Universitätsmedizin Berlin, corporate member of Freie Universität Berlin, Humboldt-Universität zu Berlin, and Berlin Institute of Health, NeuroCure Clinical Research Center, Berlin, Germany
[30]Research Institute of Medical Genetics of Russian Academy of Medical Sciences, Russian Federation
[31]Cold Spring Harbor Laboratory, Woodbury, United States
[32]University of Bonn, Bonn, Germany
[33]Hospital General Universitario De Valencia, Valencia, Spain
[34]Hospital General De Requena, Servicio Pediatría, Spain
[35]University Hospital Leipzig, Leipzig, Germany
[36]Hospital Universitario Miguel Servet, Spain
[37]Azienda Ospedaliera Universitaria Senese, Siena, Italy
[38]Children's Hospital Colorado, United States
[39]Clinical Institute of Medical Genetics, University Medical Centre Ljubljana, Ljubljana, Slovenia
[40]Sheba Medical Center, Israel
[41]Université Catholique de Louvain, Bruxelles, Belgium
[42]Odense University Hospital, Odense, Denmark
[43]The Jackson Laboratory for Genomic Medicine, Farmington, United States
[44]School of Medicine, University of Leeds, Leeds, United Kingdom
[45]Ariel University, Ariel, Israel
[46]Center for Prenatal Diagnosis and Human Genetics, Berlin, Germany
[47]Cliniques universitaires Saint Luc UCL, Bruxelles, Belgium
[48]Institute of Human Genetics, Friedrich-Alexander-Universität Erlangen-Nürnberg FAU, Erlangen, Erlangen, Germany
[49]Hopital Robert Debré, Paris, France
[50]Center for Human Genetics and Laboratory Diagnostics, Germany
[51]Poznañ University of Medical Sciences, Poznañ, Poland
[52]University Medical Center Göttingen, Göttingen, Germany
[*]equally contributing first authors
[+]corresponding author, pkrawitz@uni-bonn.de

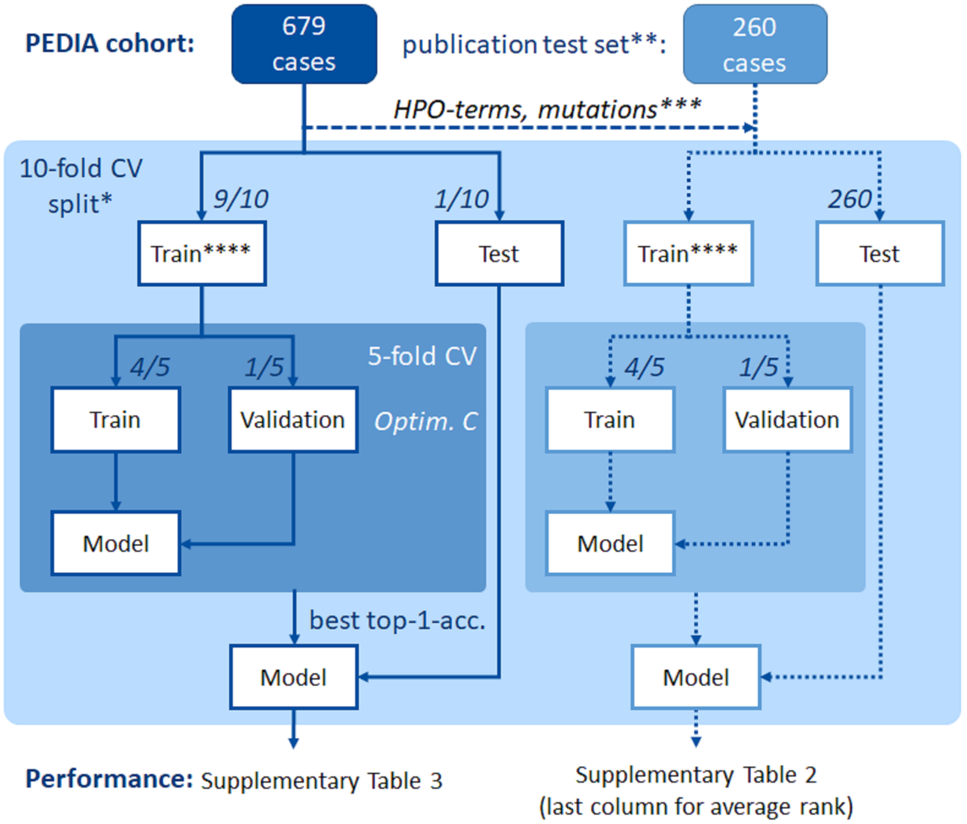

Supplementary Figure 1: Training flowchart. (CV: cross-validation; optim.: optimizing; top-1-acc: top-1-accuracy; *cases with the same disease-causing gene were sampled to one bin; **from DeepGestalt, contains only photos, labled with diagnosis; ***random assignment of a photo to a matching case from PEDIA cohort to test set. The cases in PEDIA cohort with the same mutations in test set were removed from training set; ****the entire data is available as json files at: `www.pedia-study.org/pedia_services/download`)

The influence of the ethnic background on the performance of the PEDIA approach was analyzed by spiking in the disease-causing mutations into the different populations of the 1000 genomes project (ACB, African Caribbeans in Barbados; ASW Americans of African Ancestry in SW USA; BEB, Bengali from Bangladesh; CDX, Chinese Dai in Xishuangbanna, China; CEU Western European Ancestry; CHBHan Chinese in Beijing, China; CHS, Southern Han Chinese; CLM, Colombians from Medellin, Colombia; ESN, Esan in Nigeria; FIN, Finnish in Finland; GBR, British in England Scotland; GIH, Gujarati Indian from Houston Texas; GWD, Gambian in Western Divisions in the Gambia; IBS, Iberian Population in Spain; ITU, Indian Telugu from the UK; JPT, Japanese in Tokyo, Japan; KHV, Kinh in Ho Chi Minh City, Vietnam; LWK, Luhya in Webuye, Kenya; MSL, Mende in Sierra Leone; MXL, Mexican Ancestry from Los Angeles USA; PEL, Peruvians from lima, Peru; PJL, Punjabi from Lahore, Pakistan; PUR, Puerto Ricans from Puerto Rico; STU, Sri Lankan Tamil from the UK; TSI, Toscani in Italia; YRI, Yoruba in Ibadan, Nigeria).

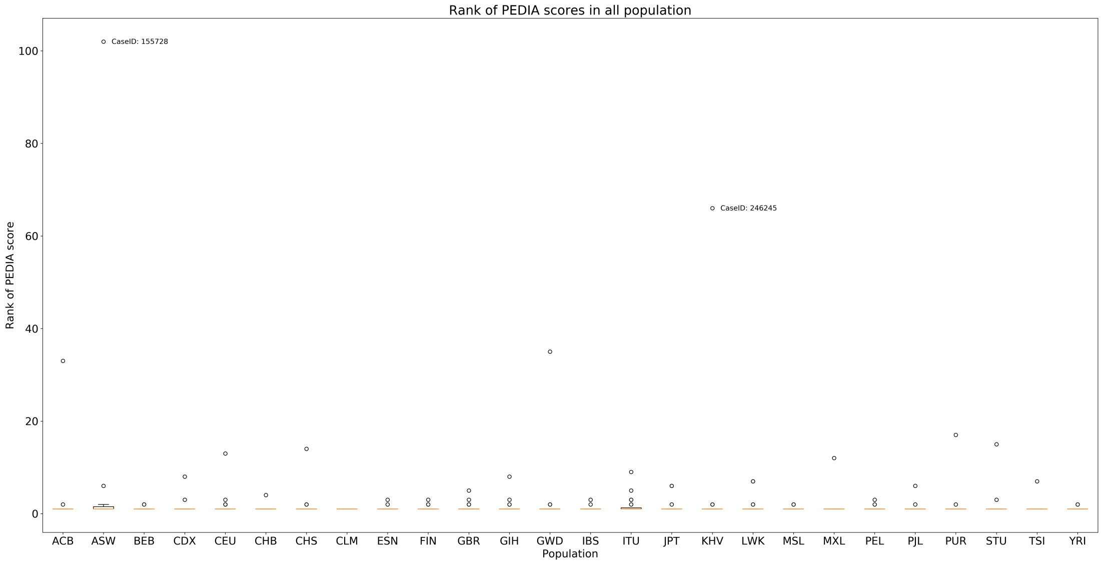

Supplementary Figure 2: The disease-causing mutations of the PEDIA cohort have been spiked into randomly selected samples from the 1KGP project. The figure shows the results of a simulation. The mean rank that the disease-causing gene achieves, does not depend on the ethnic background. In two cases the performance is exceptionally poor, due to a low CADD score of the pathogenic variant (case ID 246245) and due to a faulty link between disorder and disease-causing gene (case ID 155728).

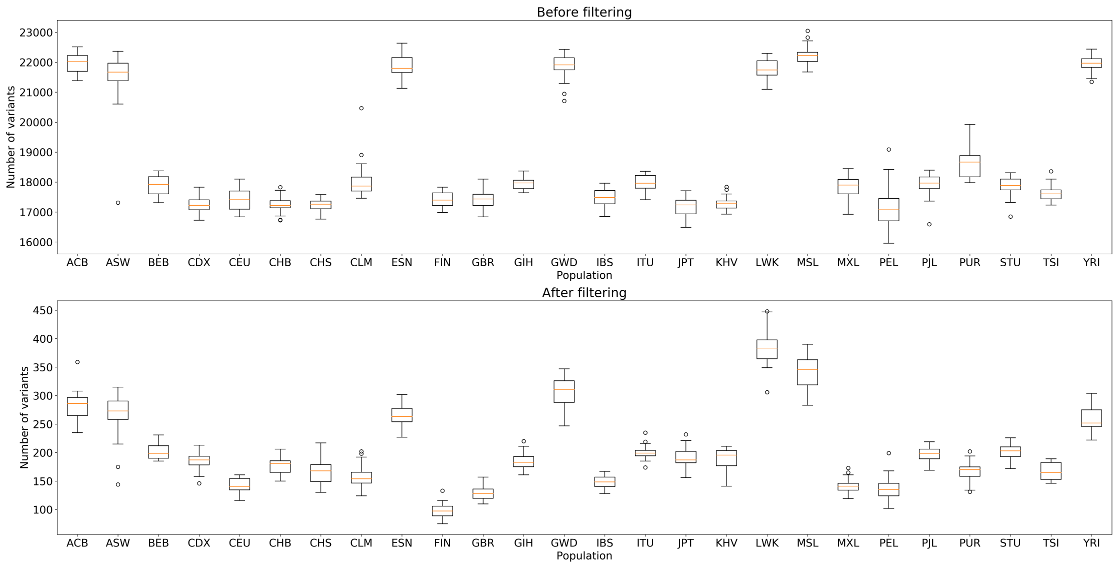

Supplementary Figure 3: The number of variants that are called in reference-guided exome sequencing varies depending on the ethnic background. Filtering according to Wright, et al., results in 100 to 400 rare variants, with the smallest numbers observed in individuals from Finland and the largest in individuals from Kenya.

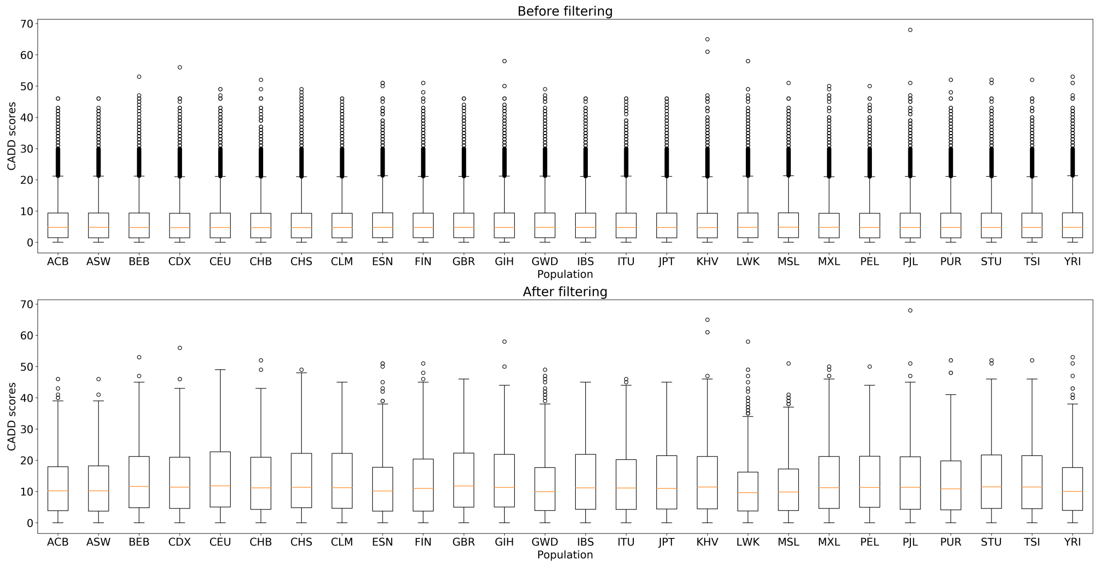

Supplementary Figure 4: The distribution of CADD scores is less dependent on the ethnic background. In all populations there is a shift in the median of the CADD scores after filtering for rare variants.

Supplementary Table 2: Comparision of results by using DeepGestalt and PEDIA on DeepGestalt publication test set. The PEDIA rank is the average rank. In order to download the images, you should first login to Face2Gene and then follow the links in the table. For any questions or requests, please contact Yaron Gurovich (yaron@fdna.com).

Index	Syndrome Name	Photo Link	DeepGestalt	PEDIA
1	Alagille Syndrome	https://app.face2gene.com/dg/lmd/110/photo/345	28	1.8
2	Nijmegen Breakage Syndrome; NBS	https://app.face2gene.com/dg/lmd/347/photo/4734	2	1.0
3	Nijmegen Breakage Syndrome; NBS	https://app.face2gene.com/dg/lmd/347/photo/4730	2	1.0
4	Craniodiaphyseal Dysplasia; CDD	https://app.face2gene.com/dg/lmd/373/photo/1088	1	1.0
5	Noonan Syndrome	https://app.face2gene.com/dg/lmd/1946/photo/5976	19	1.4
6	Noonan Syndrome	https://app.face2gene.com/dg/lmd/1946/photo/5985	11	1.1
7	Smith-Lemli-Opitz Syndrome; SLOS	https://app.face2gene.com/dg/lmd/1608/photo/4893	1	1.0
8	Kabuki Syndrome	https://app.face2gene.com/dg/lmd/893/photo/2567	1	1.0
9	Focal Facial Dermal Dysplasia 3, Setleis Type; FFDD3	https://app.face2gene.com/dg/lmd/1570/photo/4760	5	1.0
10	Burn-Mckeown Syndrome; BMKS	https://app.face2gene.com/dg/lmd/2907/photo/6717	1	1.0
11	Burn-Mckeown Syndrome; BMKS	https://app.face2gene.com/dg/lmd/2907/photo/6716	1	1.0
12	Burn-Mckeown Syndrome; BMKS	https://app.face2gene.com/dg/lmd/2907/photo/6719	18	2.8
13	Burn-Mckeown Syndrome; BMKS	https://app.face2gene.com/dg/lmd/2907/photo/6720	1	1.0
14	Meier-Gorlin Syndrome 1; MGORS1	https://app.face2gene.com/dg/lmd/808/photo/2269	30	1.2
16	Trichorhinophalangeal Syndrome, Type II; TRPS2	https://app.face2gene.com/dg/lmd/971/photo/2778	1	1.0
17	Acrocallosal Syndrome; ACLS	https://app.face2gene.com/dg/lmd/23/photo/80	7	1.6
18	Cohen Syndrome; COH1	https://app.face2gene.com/dg/lmd/333/photo/1003	1	1.0
19	Schwartz-Jampel Syndrome, Type 1; SJS1	https://app.face2gene.com/dg/lmd/1548/photo/4685	9	1.0
20	Treacher Collins Syndrome 1; TCS1	https://app.face2gene.com/dg/lmd/1720/photo/5245	1	1.0
21	Trichorhinophalangeal Syndrome	https://app.face2gene.com/dg/lmd/1730/photo/5290	1	1.0
22	Velocardiofacial Syndrome	https://app.face2gene.com/dg/lmd/1762/photo/8285	3	1.0
23	Smith-Lemli-Opitz Syndrome; SLOS	https://app.face2gene.com/dg/lmd/1608/photo/8342	1	1.0
24	Marfan Syndrome; MFS	https://app.face2gene.com/dg/lmd/1057/photo/8255	1	1.0
26	Mowat-Wilson Syndrome; MOWS	https://app.face2gene.com/dg/lmd/4211/photo/13183	1	1.0
27	Mowat-Wilson Syndrome; MOWS	https://app.face2gene.com/dg/lmd/4211/photo/13743	1	1.0
28	Mowat-Wilson Syndrome; MOWS	https://app.face2gene.com/dg/lmd/4211/photo/13744	1	1.0
29	3MC Syndrome 3; 3MC3	https://app.face2gene.com/dg/lmd/1048/photo/14730	2	1.0
30	Sotos Syndrome 2; SOTOS2	https://app.face2gene.com/dg/lmd/1617/photo/15149	1	1.1

31	Sotos Syndrome 2; SOTOS2	https://app.face2gene.com/dg/lmd/1617/photo/15152	1	1.0
32	Treacher Collins Syndrome 1; TCS1	https://app.face2gene.com/dg/lmd/1720/photo/5227	1	1.0
33	Coffin-Lowry Syndrome; CLS	https://app.face2gene.com/dg/lmd/328/photo/958	1	1.0
35	Mowat-Wilson Syndrome; MOWS	https://app.face2gene.com/dg/lmd/4211/photo/16768	1	1.0
36	Donnai-Barrow Syndrome	https://app.face2gene.com/dg/lmd/3473/photo/16950	49	1.2
37	Floating-Harbor Syndrome; FLHS	https://app.face2gene.com/dg/lmd/596/photo/17148	17	1.2
38	Pitt-Hopkins Syndrome; PTHS	https://app.face2gene.com/dg/lmd/8892/photo/17151	1	1.0
39	Marshall-Smith Syndrome; MRSHSS	https://app.face2gene.com/dg/lmd/7513/photo/17204	1	1.0
40	3MC Syndrome 3; 3MC3	https://app.face2gene.com/dg/lmd/1048/photo/17445	110	1.9
42	Waardenburg Syndrome, Type 1; WS1	https://app.face2gene.com/dg/lmd/1781/photo/5438	37	1.0
44	Sotos Syndrome 2; SOTOS2	https://app.face2gene.com/dg/lmd/1617/photo/4945	1	1.0
45	Pitt-Hopkins Syndrome; PTHS	https://app.face2gene.com/dg/lmd/8892/photo/17999	1	1.0
46	Kabuki Syndrome	https://app.face2gene.com/dg/lmd/893/photo/18077	1	1.0
47	Waardenburg Syndrome, Type 1; WS1	https://app.face2gene.com/dg/lmd/1781/photo/8786	1	1.0
48	Waardenburg Syndrome, Type 1; WS1	https://app.face2gene.com/dg/lmd/1781/photo/8794	25	1.0
49	Smith-Magenis Syndrome; SMS	https://app.face2gene.com/dg/lmd/3562/photo/9086	1	1.0
51	Seckel Syndrome	https://app.face2gene.com/dg/lmd/739/photo/9429	2	1.0
52	Rothmund-Thomson Syndrome; RTS	https://app.face2gene.com/dg/lmd/1487/photo/9782	66	1.4
54	Coffin-Siris Syndrome1; CSS1	https://app.face2gene.com/dg/lmd/329/photo/18535	15	1.0
55	Bohring-Opitz Syndrome; BOPS	https://app.face2gene.com/dg/lmd/4337/photo/18830	1	1.0
56	SHORT syndrome	https://app.face2gene.com/dg/lmd/1574/photo/4787	1	1.0
57	Schwartz-Jampel Syndrome, Type 1; SJS1	https://app.face2gene.com/dg/lmd/1548/photo/4686	4	1.0
58	Hajdu-Cheney Syndrome; HJCYS	https://app.face2gene.com/dg/lmd/722/photo/2067	1	1.0
59	Coffin-Lowry Syndrome; CLS	https://app.face2gene.com/dg/lmd/328/photo/967	1	1.0
60	Baraitser-Winter Syndrome 1; BRWS1	https://app.face2gene.com/dg/lmd/149/photo/430	2	1.0
61	Beckwith-Wiedemann Syndrome; BWS	https://app.face2gene.com/dg/lmd/173/photo/480	3	1.0
62	Baraitser-Winter Syndrome 1; BRWS1	https://app.face2gene.com/dg/lmd/149/photo/432	2	1.0
66	Sotos Syndrome 2; SOTOS2	https://app.face2gene.com/dg/lmd/1617/photo/4944	1	1.0
67	Cockayne Syndrome	https://app.face2gene.com/dg/lmd/326/photo/946	1	1.0
68	Acromicric Dysplasia	https://app.face2gene.com/dg/lmd/30/photo/145	24	1.0
70	Ectodermal Dysplasia 1, Hypohidrotic, X-Linked; XHED	https://app.face2gene.com/dg/lmd/508/photo/1407	1	1.0
71	Coffin-Lowry Syndrome; CLS	https://app.face2gene.com/dg/lmd/328/photo/960	6	1.0

72	Mucolipidosis II Alpha/beta	https://app.face2gene.com/dg/lmd/838/photo/2386	5	1.0
73	Beckwith-Wiedemann Syndrome; BWS	https://app.face2gene.com/dg/lmd/173/photo/484	6	1.0
74	Craniodiaphyseal Dysplasia; CDD	https://app.face2gene.com/dg/lmd/373/photo/1093	2	1.0
75	Cockayne Syndrome	https://app.face2gene.com/dg/lmd/326/photo/948	3	1.0
76	Floating-Harbor Syndrome; FLHS	https://app.face2gene.com/dg/lmd/596/photo/1761	1	1.0
77	Rubinstein-Taybi Syndrome 1; RSTS1	https://app.face2gene.com/dg/lmd/1490/photo/4474	1	1.0
78	Coffin-Lowry Syndrome; CLS	https://app.face2gene.com/dg/lmd/328/photo/970	1	1.0
79	Coffin-Lowry Syndrome; CLS	https://app.face2gene.com/dg/lmd/328/photo/971	1	1.0
80	Apert Syndrome	https://app.face2gene.com/dg/lmd/99/photo/326	1	1.0
81	Branchiooculofacial Syndrome; BOFS	https://app.face2gene.com/dg/lmd/633/photo/8345	1	1.0
82	Smith-Lemli-Opitz Syndrome; SLOS	https://app.face2gene.com/dg/lmd/1608/photo/12105	1	1.0
83	Smith-Lemli-Opitz Syndrome; SLOS	https://app.face2gene.com/dg/lmd/1608/photo/12105	1	1.0
84	Coffin-Siris Syndrome1; CSS1	https://app.face2gene.com/dg/lmd/329/photo/12535	3	1.0
85	Coffin-Siris Syndrome1; CSS1	https://app.face2gene.com/dg/lmd/329/photo/12535	2	1.0
86	Kabuki Syndrome	https://app.face2gene.com/dg/lmd/893/photo/13114	1	1.0
87	Kabuki Syndrome	https://app.face2gene.com/dg/lmd/893/photo/13116	1	1.0
88	Mowat-Wilson Syndrome; MOWS	https://app.face2gene.com/dg/lmd/4211/photo/13734	1	1.0
89	Mowat-Wilson Syndrome; MOWS	https://app.face2gene.com/dg/lmd/4211/photo/13736	58	1.5
90	Mowat-Wilson Syndrome; MOWS	https://app.face2gene.com/dg/lmd/4211/photo/13742	1	1.0
91	Borjeson-Forssman-Lehmann Syndrome; BFLS	https://app.face2gene.com/dg/lmd/216/photo/14759	56	2.8
92	Smith-Magenis Syndrome; SMS	https://app.face2gene.com/dg/lmd/3562/photo/14969	4	1.4
95	Opitz GBBB Syndrome, Type II; GBBB2	https://app.face2gene.com/dg/lmd/640/photo/15940	9	1.0
98	Sotos Syndrome 2; SOTOS2	https://app.face2gene.com/dg/lmd/1617/photo/17023	1	1.0
99	Sotos Syndrome 2; SOTOS2	https://app.face2gene.com/dg/lmd/1617/photo/17024	10	1.2
100	CHARGE Syndrome	https://app.face2gene.com/dg/lmd/301/photo/17094	2	1.0
101	Floating-Harbor Syndrome; FLHS	https://app.face2gene.com/dg/lmd/596/photo/17124	1	1.0
103	Craniometaphyseal Dysplasia	https://app.face2gene.com/dg/lmd/371/photo/7667	22	1.0
105	Pitt-Hopkins Syndrome; PTHS	https://app.face2gene.com/dg/lmd/8892/photo/18000	1	1.0
106	Baraitser-Winter Syndrome 1; BRWS1	https://app.face2gene.com/dg/lmd/149/photo/10154	10	1.3
107	Baraitser-Winter Syndrome 1; BRWS1	https://app.face2gene.com/dg/lmd/149/photo/10156	41	2.0
108	Baraitser-Winter Syndrome 1; BRWS1	https://app.face2gene.com/dg/lmd/149/photo/10156	41	1.9
109	3MC Syndrome 3; 3MC3	https://app.face2gene.com/dg/lmd/1048/photo/10305	2	1.0

111	Smith-Magenis Syndrome; SMS	https://app.face2gene.com/dg/lmd/3562/photo/18744	1	1.0
112	Smith-Magenis Syndrome; SMS	https://app.face2gene.com/dg/lmd/3562/photo/18745	4	1.0
113	Smith-Magenis Syndrome; SMS	https://app.face2gene.com/dg/lmd/3562/photo/18747	1	1.5
114	Smith-Magenis Syndrome; SMS	https://app.face2gene.com/dg/lmd/3562/photo/18749	1	1.1
118	Crouzon Syndrome	https://app.face2gene.com/dg/lmd/383/photo/1133	1	1.0
120	Beckwith-Wiedemann Syndrome; BWS	https://app.face2gene.com/dg/lmd/173/photo/41779	1	1.0
121	Otopalatodigital Syndrome	https://app.face2gene.com/dg/lmd/1266/photo/41780	1	1.0
122	Waardenburg Syndrome, Type 1; WS1	https://app.face2gene.com/dg/lmd/1781/photo/41781	1	1.0
123	Rubinstein-Taybi Syndrome	https://app.face2gene.com/dg/lmd/1490/photo/41785	1	1.0
124	Treacher Collins Syndrome	https://app.face2gene.com/dg/lmd/1720/photo/41799	1	1.0
125	Cornelia De Lange Syndrome	https://app.face2gene.com/dg/lmd/423/photo/41811	1	1.5
126	Cornelia De Lange Syndrome	https://app.face2gene.com/dg/lmd/423/photo/41812	1	1.5
127	Cornelia De Lange Syndrome	https://app.face2gene.com/dg/lmd/423/photo/41813	1	1.5
128	Cornelia De Lange Syndrome	https://app.face2gene.com/dg/lmd/423/photo/41814	1	1.5
129	Sotos Syndrome	https://app.face2gene.com/dg/lmd/1617/photo/41817	1	1.1
130	Ectodermal Dysplasia 1, Hypohidrotic, X-Linked; XHED	https://app.face2gene.com/dg/lmd/508/photo/41823	2	1.0
134	Hutchinson-Gilford Progeria Syndrome; HGPS	https://app.face2gene.com/dg/lmd/1396/photo/41849	1	1.0
136	Hurler Syndrome	https://app.face2gene.com/dg/lmd/13858/photo/41873	1	1.1
137	Hurler Syndrome	https://app.face2gene.com/dg/lmd/13858/photo/41874	1	1.1
138	Mucopolysaccharidosis, Type II; MPS2	https://app.face2gene.com/dg/lmd/801/photo/41875	1	1.1
139	Mucopolysaccharidosis, Type II; MPS2	https://app.face2gene.com/dg/lmd/801/photo/41876	1	1.0
140	Mucopolysaccharidosis, Type II; MPS2	https://app.face2gene.com/dg/lmd/801/photo/41878	1	1.1
141	Mucopolysaccharidosis, Type II; MPS2	https://app.face2gene.com/dg/lmd/801/photo/41879	1	1.1
142	Mucolipidosis II Alpha/beta	https://app.face2gene.com/dg/lmd/838/photo/41883	1	1.0
143	Cleidocranial Dysplasia; CCD	https://app.face2gene.com/dg/lmd/13331/photo/41890	5	1.0
144	Cleidocranial Dysplasia; CCD	https://app.face2gene.com/dg/lmd/13331/photo/41891	1	1.0
145	Seckel Syndrome	https://app.face2gene.com/dg/lmd/739/photo/41904	12	1.2
147	Crouzon Syndrome	https://app.face2gene.com/dg/lmd/383/photo/41914	2	1.0
153	Sotos Syndrome	https://app.face2gene.com/dg/lmd/1617/photo/41940	1	1.0
154	Sotos Syndrome	https://app.face2gene.com/dg/lmd/1617/photo/41941	1	1.2
157	Cleidocranial Dysplasia; CCD	https://app.face2gene.com/dg/lmd/13331/photo/41947	1	1.0
158	Treacher Collins Syndrome	https://app.face2gene.com/dg/lmd/1720/photo/41956	4	1.0

159	Treacher Collins Syndrome	https://app.face2gene.com/dg/lmd/1720/photo/41958	1	1.0
163	Fragile X Mental Retardation Syndrome	https://app.face2gene.com/dg/lmd/13243/photo/41973	1	3.3
165	Smith-Magenis Syndrome; SMS	https://app.face2gene.com/dg/lmd/3562/photo/15947	1	1.4
166	Pierpont Syndrome; PRPTS	https://app.face2gene.com/dg/lmd/4198/photo/41638	3	1.0
167	Pierpont Syndrome; PRPTS	https://app.face2gene.com/dg/lmd/4198/photo/41640	1	1.0
168	SHORT syndrome	https://app.face2gene.com/dg/lmd/1574/photo/4780	1	1.0
169	Kabuki Syndrome	https://app.face2gene.com/dg/lmd/893/photo/2566	1	1.0
170	Kabuki Syndrome	https://app.face2gene.com/dg/lmd/893/photo/2560	1	1.0
171	Kabuki Syndrome	https://app.face2gene.com/dg/lmd/893/photo/2562	8	1.8
172	Otopalatodigital Syndrome	https://app.face2gene.com/dg/lmd/1266/photo/3719	1	1.0
173	Coffin-Lowry Syndrome; CLS	https://app.face2gene.com/dg/lmd/328/photo/977	1	1.0
174	Ohdo Syndrome, SBBYS Variant; SBBYSS	https://app.face2gene.com/dg/lmd/1255/photo/8343	1	1.0
175	Rubinstein-Taybi Syndrome 1; RSTS1	https://app.face2gene.com/dg/lmd/358/photo/11625	2	1.1
176	Auriculocondylar Syndrome 1; ARCND1	https://app.face2gene.com/dg/lmd/4192/photo/12354	1	1.0
177	Coffin-Siris Syndrome1; CSS1	https://app.face2gene.com/dg/lmd/329/photo/12530	1	1.0
178	Urofacial Syndrome 1; UFS1	https://app.face2gene.com/dg/lmd/1236/photo/13084	3	1.0
179	3MC Syndrome 3; 3MC3	https://app.face2gene.com/dg/lmd/1048/photo/13477	1	1.0
180	Smith-Lemli-Opitz Syndrome; SLOS	https://app.face2gene.com/dg/lmd/1608/photo/14313	1	1.0
181	3MC Syndrome 3; 3MC3	https://app.face2gene.com/dg/lmd/1048/photo/14727	1	1.0
183	Lig4 Syndrome	https://app.face2gene.com/dg/lmd/4654/photo/17338	16	1.0
184	Craniometaphyseal Dysplasia	https://app.face2gene.com/dg/lmd/371/photo/1078	1	1.0
188	Craniofrontonasal Syndrome; CFNS	https://app.face2gene.com/dg/lmd/376/photo/1113	1	1.0
190	Floating-Harbor Syndrome; FLHS	https://app.face2gene.com/dg/lmd/596/photo/1756	1	1.0
191	Rubinstein-Taybi Syndrome 1; RSTS1	https://app.face2gene.com/dg/lmd/1490/photo/4488	1	1.0
192	Coffin-Siris Syndrome1; CSS1	https://app.face2gene.com/dg/lmd/329/photo/981	1	1.0
193	Rubinstein-Taybi Syndrome 1; RSTS1	https://app.face2gene.com/dg/lmd/1490/photo/4489	1	1.0
194	Noonan Syndrome	https://app.face2gene.com/dg/lmd/1229/photo/3585	1	1.1
195	Floating-Harbor Syndrome; FLHS	https://app.face2gene.com/dg/lmd/596/photo/8231	1	1.0
196	Smith-Lemli-Opitz Syndrome; SLOS	https://app.face2gene.com/dg/lmd/1608/photo/12105	1	1.0
197	Smith-Lemli-Opitz Syndrome; SLOS	https://app.face2gene.com/dg/lmd/1608/photo/12105	2	1.0
198	Kabuki Syndrome	https://app.face2gene.com/dg/lmd/893/photo/13113	1	1.0
199	Kabuki Syndrome	https://app.face2gene.com/dg/lmd/893/photo/13113	1	1.1
200	Sotos Syndrome 2; SOTOS2	https://app.face2gene.com/dg/lmd/1617/photo/15150	1	1.1
203	Sotos Syndrome 2; SOTOS2	https://app.face2gene.com/dg/lmd/1617/photo/17025	1	1.1

205	Smith-Magenis Syndrome; SMS	https://app.face2gene.com/dg/lmd/3562/photo/18745	1	1.3
206	Smith-Magenis Syndrome; SMS	https://app.face2gene.com/dg/lmd/3562/photo/18745	1	1.1
207	Birk-Barel Mental Retardation Dysmorphism Syndrome	https://app.face2gene.com/dg/lmd/5569/photo/19317	12	1.4
209	Focal Dermal Hypoplasia; FDH	https://app.face2gene.com/dg/lmd/13389/photo/41764	1	1.0
214	Cornelia De Lange Syndrome	https://app.face2gene.com/dg/lmd/423/photo/41808	1	1.0
215	Cornelia De Lange Syndrome	https://app.face2gene.com/dg/lmd/423/photo/41810	1	1.0
216	Sotos Syndrome	https://app.face2gene.com/dg/lmd/1617/photo/41818	1	1.2
218	Focal Dermal Hypoplasia; FDH	https://app.face2gene.com/dg/lmd/13389/photo/41822	6	1.0
219	Mucopolysaccharidosis, Type II; MPS2	https://app.face2gene.com/dg/lmd/801/photo/41824	3	1.2
221	Crouzon Syndrome	https://app.face2gene.com/dg/lmd/383/photo/41842	1	1.0
222	Rubinstein-Taybi Syndrome	https://app.face2gene.com/dg/lmd/1490/photo/41844	1	1.0
223	Hutchinson-Gilford Progeria Syndrome; HGPS	https://app.face2gene.com/dg/lmd/1396/photo/41845	1	1.0
224	Hutchinson-Gilford Progeria Syndrome; HGPS	https://app.face2gene.com/dg/lmd/1396/photo/41846	1	1.0
225	Hutchinson-Gilford Progeria Syndrome; HGPS	https://app.face2gene.com/dg/lmd/1396/photo/41847	1	1.0
226	Hutchinson-Gilford Progeria Syndrome; HGPS	https://app.face2gene.com/dg/lmd/1396/photo/41848	1	1.0
227	Hutchinson-Gilford Progeria Syndrome; HGPS	https://app.face2gene.com/dg/lmd/1396/photo/41850	1	1.0
228	Noonan Syndrome	https://app.face2gene.com/dg/lmd/1229/photo/41851	1	1.0
231	Rubinstein-Taybi Syndrome	https://app.face2gene.com/dg/lmd/1490/photo/41859	1	1.0
232	Achondroplasia; ACH	https://app.face2gene.com/dg/lmd/17/photo/41871	9	1.0
233	Hurler Syndrome	https://app.face2gene.com/dg/lmd/13858/photo/41872	1	1.4
234	Cleidocranial Dysplasia; CCD	https://app.face2gene.com/dg/lmd/13331/photo/41899	1	1.0
236	Saethre-Chotzen Syndrome; SCS	https://app.face2gene.com/dg/lmd/1504/photo/41909	1	1.0
237	Cleidocranial Dysplasia; CCD	https://app.face2gene.com/dg/lmd/13331/photo/41948	7	1.6
238	Treacher Collins Syndrome	https://app.face2gene.com/dg/lmd/1720/photo/41951	1	1.0
239	Treacher Collins Syndrome	https://app.face2gene.com/dg/lmd/1720/photo/41952	1	1.0
240	Treacher Collins Syndrome	https://app.face2gene.com/dg/lmd/1720/photo/41954	1	1.0
241	Treacher Collins Syndrome	https://app.face2gene.com/dg/lmd/1720/photo/41959	1	1.0
242	Treacher Collins Syndrome	https://app.face2gene.com/dg/lmd/1720/photo/41960	1	1.0
243	Fragile X Mental Retardation Syndrome	https://app.face2gene.com/dg/lmd/13243/photo/41982	1	12.6

244	Pitt-Hopkins Syndrome; PTHS	https://app.face2gene.com/dg/lmd/8892/photo/18003	1	1.0
245	Nijmegen Breakage Syndrome; NBS	https://app.face2gene.com/dg/lmd/347/photo/4723	1	1.0
246	Nijmegen Breakage Syndrome; NBS	https://app.face2gene.com/dg/lmd/347/photo/4736	13	1.0
247	Noonan Syndrome	https://app.face2gene.com/dg/lmd/1946/photo/5983	2	1.0
248	Kabuki Syndrome	https://app.face2gene.com/dg/lmd/893/photo/2565	1	1.1
249	Craniodiaphyseal Dysplasia; CDD	https://app.face2gene.com/dg/lmd/373/photo/1091	1	1.0
250	Ectodermal Dysplasia 1, Hypohidrotic, X-Linked; XHED	https://app.face2gene.com/dg/lmd/508/photo/1400	1	1.0
251	Mowat-Wilson Syndrome; MOWS	https://app.face2gene.com/dg/lmd/4211/photo/13735	1	1.0
252	3MC Syndrome 3; 3MC3	https://app.face2gene.com/dg/lmd/1048/photo/13467	1	1.0
253	Auriculocondylar Syndrome 1; ARCND1	https://app.face2gene.com/dg/lmd/4192/photo/14125	2	1.0
254	Nicolaides-Baraitser Syndrome; NCBRS	https://app.face2gene.com/dg/lmd/3394/photo/14255	1	1.0
255	Smith-Magenis Syndrome; SMS	https://app.face2gene.com/dg/lmd/3562/photo/14454	1	1.1
256	Nicolaides-Baraitser Syndrome; NCBRS	https://app.face2gene.com/dg/lmd/3394/photo/14700	1	1.0
257	Noonan Syndrome	https://app.face2gene.com/dg/lmd/1229/photo/3581	1	1.0
258	KBG Syndrome; KBGS	https://app.face2gene.com/dg/lmd/912/photo/15604	1	1.0
261	Beckwith-Wiedemann Syndrome; BWS	https://app.face2gene.com/dg/lmd/173/photo/481	6	1.0
262	Craniofrontonasal Syndrome; CFNS	https://app.face2gene.com/dg/lmd/376/photo/1115	1	1.0
263	Floating-Harbor Syndrome; FLHS	https://app.face2gene.com/dg/lmd/596/photo/1760	1	1.0
264	Coffin-Lowry Syndrome; CLS	https://app.face2gene.com/dg/lmd/328/photo/974	1	1.0
265	Sotos Syndrome 2; SOTOS2	https://app.face2gene.com/dg/lmd/1617/photo/15146	1	1.0
266	Smith-Magenis Syndrome; SMS	https://app.face2gene.com/dg/lmd/3562/photo/15950	1	1.2
268	Lateral Meningocele Syndrome; LMNS	https://app.face2gene.com/dg/lmd/983/photo/10028	3	1.0
269	KBG Syndrome; KBGS	https://app.face2gene.com/dg/lmd/912/photo/10163	1	1.0
270	Smith-Magenis Syndrome; SMS	https://app.face2gene.com/dg/lmd/3562/photo/18749	4	18.7
272	Bardet-Biedl Syndrome	https://app.face2gene.com/dg/lmd/13278/photo/41796	1	1.0
273	Cornelia De Lange Syndrome	https://app.face2gene.com/dg/lmd/423/photo/41807	1	1.5
275	Crouzon Syndrome	https://app.face2gene.com/dg/lmd/383/photo/41828	1	1.0
277	Rubinstein-Taybi Syndrome	https://app.face2gene.com/dg/lmd/1490/photo/41858	1	1.0
278	Rubinstein-Taybi Syndrome	https://app.face2gene.com/dg/lmd/1490/photo/41860	1	1.0
279	Mucopolysaccharidosis, Type II; MPS2	https://app.face2gene.com/dg/lmd/801/photo/41877	1	1.0
280	Trichorhinophalangeal Syndrome	https://app.face2gene.com/dg/lmd/1730/photo/41886	1	1.0
281	Trichorhinophalangeal Syndrome	https://app.face2gene.com/dg/lmd/1730/photo/41887	1	1.0
282	Cleidocranial Dysplasia; CCD	https://app.face2gene.com/dg/lmd/13331/photo/41900	32	3.1

283	Saethre-Chotzen Syndrome; SCS	https://app.face2gene.com/dg/lmd/1504/photo/41910	42	1.0
284	Treacher Collins Syndrome	https://app.face2gene.com/dg/lmd/1720/photo/41961	1	1.0
286	Frontometaphyseal Dysplasia; FMD	https://app.face2gene.com/dg/lmd/622/photo/41685	1	1.0
287	Kabuki Syndrome	https://app.face2gene.com/dg/lmd/893/photo/2569	1	1.0
288	Nijmegen Breakage Syndrome; NBS	https://app.face2gene.com/dg/lmd/347/photo/4728	7	1.0
289	Coffin-Lowry Syndrome; CLS	https://app.face2gene.com/dg/lmd/328/photo/978	1	1.0
290	Hutchinson-Gilford Progeria Syndrome; HGPS	https://app.face2gene.com/dg/lmd/1396/photo/4126	1	1.0
291	Costello Syndrome; CSTLO	https://app.face2gene.com/dg/lmd/2014/photo/6166	2	1.0
292	Kabuki Syndrome	https://app.face2gene.com/dg/lmd/893/photo/13115	1	1.0
293	Noonan Syndrome	https://app.face2gene.com/dg/lmd/4716/photo/14088	7	2.4
294	Noonan Syndrome	https://app.face2gene.com/dg/lmd/4716/photo/14090	3	1.2
295	Noonan Syndrome	https://app.face2gene.com/dg/lmd/4716/photo/14091	2	1.0
296	Noonan Syndrome	https://app.face2gene.com/dg/lmd/4716/photo/14092	1	1.0
297	KBG Syndrome; KBGS	https://app.face2gene.com/dg/lmd/912/photo/15603	1	1.0
298	Opitz GBBB Syndrome, Type II; GBBB2	https://app.face2gene.com/dg/lmd/640/photo/15945	3	1.0
299	Gapo Syndrome	https://app.face2gene.com/dg/lmd/642/photo/16435	3	1.0
300	Velocardiofacial Syndrome	https://app.face2gene.com/dg/lmd/1762/photo/19049	13	1.0
301	Velocardiofacial Syndrome	https://app.face2gene.com/dg/lmd/1762/photo/5395	21	1.0
302	Trichorhinophalangeal Syndrome	https://app.face2gene.com/dg/lmd/1730/photo/5284	1	1.0
304	Noonan Syndrome	https://app.face2gene.com/dg/lmd/1229/photo/3582	26	2.6
305	Craniofrontonasal Syndrome; CFNS	https://app.face2gene.com/dg/lmd/376/photo/1114	1	1.1
306	Trichorhinophalangeal Syndrome	https://app.face2gene.com/dg/lmd/1730/photo/5282	1	1.0
307	Alagille Syndrome	https://app.face2gene.com/dg/lmd/110/photo/349	1	1.0
308	Smith-Lemli-Opitz Syndrome; SLOS	https://app.face2gene.com/dg/lmd/1608/photo/12105	2	1.0
309	Opitz GBBB Syndrome, Type II; GBBB2	https://app.face2gene.com/dg/lmd/640/photo/15943	1	1.0
312	Cornelia de Lange Syndrome 1; CDLS1	https://app.face2gene.com/dg/lmd/423/photo/17342	1	1.5
313	Craniometaphyseal Dysplasia	https://app.face2gene.com/dg/lmd/371/photo/7667	5	1.0
314	Coffin-Lowry Syndrome; CLS	https://app.face2gene.com/dg/lmd/328/photo/17870	1	1.0
315	Coffin-Lowry Syndrome; CLS	https://app.face2gene.com/dg/lmd/328/photo/17868	6	1.0
316	Coffin-Lowry Syndrome; CLS	https://app.face2gene.com/dg/lmd/328/photo/17868	60	4.6
317	Branchiooculofacial Syndrome; BOFS	https://app.face2gene.com/dg/lmd/633/photo/9227	17	1.1
318	Smith-Magenis Syndrome; SMS	https://app.face2gene.com/dg/lmd/3562/photo/18747	2	4.7
319	Oculodentodigital Dysplasia	https://app.face2gene.com/dg/lmd/1246/photo/19085	8	1.0
320	Birk-Barel Mental Retardation Dysmorphism Syndrome	https://app.face2gene.com/dg/lmd/5569/photo/19317	15	1.0

322	Craniometaphyseal Dysplasia	https://app.face2gene.com/dg/lmd/371/photo/41763	1	1.0
323	Rubinstein-Taybi Syndrome	https://app.face2gene.com/dg/lmd/1490/photo/41843	1	1.0
324	Seckel Syndrome	https://app.face2gene.com/dg/lmd/739/photo/41855	1	1.0
326	Crouzon Syndrome	https://app.face2gene.com/dg/lmd/383/photo/41942	1	1.0
327	Treacher Collins Syndrome	https://app.face2gene.com/dg/lmd/1720/photo/41957	1	1.0
329	Frontometaphyseal Dysplasia; FMD	https://app.face2gene.com/dg/lmd/622/photo/41569	1	1.0

Supplementary Table 3: Comparision results of using different scores for gene prioritization. First column indicates the scores used in each row. The second and third columns are the Top-1 and Top-10 accuracy and confidance interval. For genes with the same PEDIA score, the highest (worst) rank was used for all of them, as this represents best the associated time for the workup. (F, Feature match score. C, CADD score. G, Gestalt score. P, Phenomizer score. B, Boqa score).

	Top-1 (%)	Top-10 (%)
**Photo + Exome + Feature**		
F,C,G,B,P	89.4 [81.4 - 97.4]	98.7 [95.7 - 100]
C,G,B,P	89.0 [80.0 - 98.0]	98.7 [95.1 - 100]
F,C,G,P	89.1 [79.9 - 98.3]	98.5 [95.1 - 100]
F,C,G,B	87.9 [78.7 - 97.1]	98.1 [94.7 - 100]
C,G,P	88.5 [79.3 - 97.7]	98.2 [93.8 - 100]
C,G,B	86.0 [75.4 - 96.6]	97.8 [95.8 - 99.8]
F,C,G	87.8 [76.4 - 99.2]	97.9 [94.1 - 100]
**Photo + Exome**		
C,G	82.6 [67.6 - 97.6]	97.5 [95.1 - 99.9]
**Photo + Feature**		
F,G,B,P	25.1 [1.7 - 48.5]	69.2 [35.8 - 100]
G,B,P	24.0 [3.0 - 45.0]	65.0 [32.4 - 97.6]
F,G,P	20.5 [1.7 - 39.3]	67.2 [33.0 - 100]
F,G,B	23.1 [1.5 - 44.7]	65.9 [36.1 - 95.7]
G,P	18.6 [2.6 - 34.6]	61.3 [24.9 - 97.7]
G,B	22.0 [4.6 - 39.4]	58.6 [31.4 - 85.8]
F,G	16.8 [0 - 34.4]	59.2 [29.8 - 88.6]
**Exome + Feature**		
F,C,B,P	74.4 [60.0 - 88.8]	93.7 [88.5 - 98.9]
C,B,P	61.4 [43.2 - 79.6]	89.0 [84.2 - 93.8]
F,C,P	74.1 [64.1 - 84.1]	93.8 [87.2 - 100]
F,C,B	65.8 [47.8 - 83.8]	91.3 [82.1 - 100]
C,P	57.9 [39.1 - 76.7]	88.7 [81.3 - 96.1]
C,B	35.5 [12.1 - 58.9]	62.8 [40.4 - 85.2]
F,C	65.1 [49.1 - 81.1]	90.9 [80.5 - 100]
**Photo**		
G	14.3 [0.3 - 28.3]	48.6 [23.4 - 73.8]
**Exome**		
C	14.1 [0 - 31.1]	44.6 [13.6 - 75.6]
**Feature**		
F,B,P	16.4 [0 - 34.2]	54.4 [25.4 - 83.4]
B,P	16.2 [0.8 - 31.6]	48.9 [21.9 - 75.9]
F,P	16.7 [0 - 36.9]	53.8 [27.0 - 80.6]
F,B	16.4 [0 - 34.4]	53.8 [24.6 - 83.0]
P	1.8 [0 - 4.6]	16.4 [0 - 33.2]
B	11.2 [0 - 28.8]	46.4 [22.0 - 70.8]
F	13.4 [0 - 29.4]	50.7 [25.5 - 75.9]
